# Zero-Material Cost Production of Soil-Coated Fabrics with Underwater Superoleophobicity for Antifouling Oil/Water Separation

**DOI:** 10.3390/membranes13030276

**Published:** 2023-02-26

**Authors:** Maohui Li, Fangfang Li, Cheng Zhen, Panpan Fu, Shaolin Yang, Youjun Lu

**Affiliations:** School of Materials Science and Engineering, International Scientific and Technological Cooperation Base of Industrial Waste Recycling and Advanced Materials, North Minzu University, Yinchuan 750021, China

**Keywords:** oil/water separation, soil, cotton fabric, underwater superoleophobicity, antifouling

## Abstract

Soil-coated fabrics were fabricated by scrape-coating of soil slurry onto cotton fabrics. The raw materials, soil, and cotton fabrics were, respectively, obtained from farmland and waste bed sheets, making the method a zero-material cost way to produce superwetting membrane. The superhydrophilic/underwater superoleophobic soil-coated fabrics exhibit high efficiency (>99%), ultra-high flux (~45,000 L m^−2^ h^−1^), and excellent antifouling behavior for separating water from various oils driven by gravity. The simple fabrication and superior performance suggest that the soil-coated fabric could be a promising candidate as a filtration membrane for practical applications in industrial oily wastewater and oil spill treatments.

## 1. Introduction

With the often occurrence of oil leakage accidents and growing industrial oily wastewater, oil/water separation has attracted much attention in recent years [1]. Possessing the advantages including easy manipulation, high efficiency, environmental friendliness, and low energy cost, membrane separation is treated as the best technique for oil pollution treatment [2,3], in comparison with traditional methods such as in situ burning [4], skimming [5], gas flotation [6], and absorption [7]. Membrane filtration was realized by a superwetting membrane, which allows the permeation of one phase but repels the other phase of the oil/water mixture.

Superhydrophobic–superoleophilic membranes have been applied to oil/water separation via discharging oil from water [8]. Yet, such “oil-removal” materials tend to be stained and even clogged by viscous oil in the successive oil permeation process, which would greatly influence the separation efficiency and recyclability. The oil density is generally lower than water in most practical oil/water separation, and the permeated oil with a lighter density would be inhibited by the water layer sandwiched between the oil phase and the superhydrophobic membrane. To overcome these obstacles, superhydrophilic–underwater superoleophobic membranes have been invented, which can effectively avert oil fouling and achieve oil/water separation through in situ oil repellence and water permeance [9].

Up to now, various membranes with underwater superoleophobicity have been produced through coating of metal oxide [10], polymers [11], graphene oxide [12], and composites [13] on porous substrates through electrodeposition, hydrothermal reaction, vacuum filtration, and spraying. For example, Song et al. prepared a superhydrophilic and underwater superoleophobic membrane based on cotton fabric decorated with TiO_2_ nanoparticles and citric acid via a micro-dissolution method [14]. Dai et al. fabricated a superhydrophilic and underwater superoleophobic nacre-like graphene oxide−calcium carbonate hybrid mesh through a layer-by-layer self-assembly on stainless steel mesh (SSM) [15]. Wang et al. reported a superhydrophilic and underwater superoleophobic cotton fabric produced by in situ surface deposition of polydopamine particles and subsequent adsorption of hydrophilic chitosan [16]. Wang et al. prepared superhydrophilic/underwater superoleophobic porous glass membranes via an in situ growth of silicone nanofilament network layers on the surface of sand particle-based sintered glass filters using vinyltrichlorosilane as a precursor, followed by grafting a thiol-functionalized of sulfobetaine by thiol-ene click chemistry [17]. Nevertheless, most of these approaches suffer from high costs, the complicated procedure, using toxic reagents, special devices, and complex chemical reactions. Besides, the water permeation fluxes of most membranes (about thousands of L m^−2^ h^−1^), which are not high enough for practical application. Therefore, it is necessary to develop a facile, cheap, and environmentally friendly route to fabricate a superhydrophilic–underwater superoleophobic membrane with a high separation efficiency and ultra-high water flux for practical oil/water separation.

Herein, we report a simple and green method to produce soil-coated fabric (SCF) with superhydrophilicity–underwater superoleophobicity via scraping soil slurry on cotton fabric (CF) followed by air-drying. The soil was dug from farmland, and the cotton fabrics were cut from waste bed sheet. Therefore, this method is a zero-material cost way to produce superwetting membrane. The SCF shows separation efficiency above 99% for separating different mixtures of oil and water. Moreover, the fabric also exhibits superior antifouling performance while maintaining nearly unchanged high oil/water separation efficiency and ultra-high flux of about 45,000 L m^−2^ h^−1^ in the whole separation process for 30 cycles.

## 2. Experimental

### 2.1. Materials

Cotton fabrics were cut from waste bed sheet. The soil was irrigation-silted soil, which was dug from the farmland of Yinchuan, China. All organic liquids were purchased from Shanghai Aladdin Biochemical Technology Co., Ltd., Shanghai, China.

### 2.2. Preparation of SCFs

Clean CF was scissored into 3 cm width strips. Soil was pulverized and sifted with a sieve (pore size: 100 μm). The sifted soil was then blended with water (m_soil_:m_water_ = 10:3) and stirred to a slurry. After scraping the soil slurry on the CF using a blade, the air-dried soil-coated fabric was repeatedly rinsed with water to remove the excessive soil until no soil particle was detached from the fabric. Finally, the obtained SCF was dried before use.

### 2.3. Oil/Water Separation

The separation experiment was conducted on a self-made setup equipped by fixing the water-prewetted SCF between two quartz tubes through pouring the of oil/water mixture (m_water_:m_oil_ = 1:1) into the top tube accompanied with collecting the permeated water with a baker below. The water and oil were weighted with an analytical balance (METTLER TOLEDO ME204, Zurich, Switzerland) with accuracy of 0.1 mg. The separation efficiency *μ* was computed using the formula, *μ = m*_2_*/m*_1_ × 100%. Here, *m*_1_ is the weight of water in the mixture, *m*_2_ is that of the collected water after separation. The water permeation flux *J* (L m^−2^ h^−1^) was calculated with the equation, *J* = *V*/(*S* × *t*), where *V* is the volume of the permeated water (L), *S* is the effective are of the SCF in contact with the oil/water mixture (m^2^), and *t* is permeation time of water (h).

### 2.4. Characterization

Contact angles (CA) was measured with an optical CA measuring instrument (POWEREACH JC200D2, Shanghai, China). Scanning electron microscopic (SEM) images were shot by a high-resolution field emission-scanning electron microscope (ZEISS SIGMA 500/VP, Oberkhien, Germany. X-ray diffraction (XRD) was carried out on an X-ray diffractometer (Rigaku RINTTTR III, Tokyo, Japan). Fourier transform infrared (FTIR) spectroscopy was conducted using a Nicolet 8700 FTIR spectrometer (Thermo Scientific, Waltham, MA, USA). 

## 3. Results and Discussion

**Figure 1:**
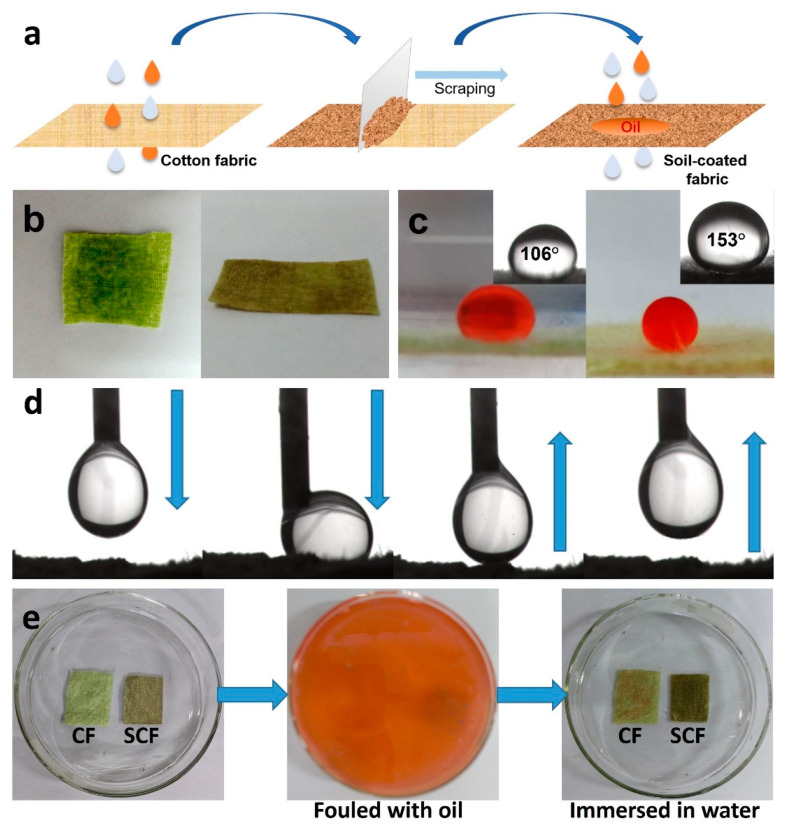
Fabrication process of the soil-coated fabric (SCF) (**a**); photos of water dripped on the cotton fabric (CF) (left) and SCF (right) (**b**); chloroform droplet on the CF (left) and SCF (right) immersed in water (inset: underwater oil CA) (**c**); contact and detachment process of chloroform droplet on the SCF surface in water (**d**); and comparison of the self-cleaning effect of CF and SCF (**e**).

As the schematic diagram shown in Figure 1a, the production process was merely realized by scrape-coating of soil slurry onto CF, demonstrating a facile, eco-friendly, and zero-material cost way to prepare superwetting membrane. Figure 1b shows the permeation of water into the CF and SCF after one droplet of water was, respectively, dripped on their surfaces, suggesting the superhydrophilic nature of both fabrics. However, the oil (chloroform) droplet shows an underwater oil CA (OCA) of 106° on the CF surface while that on SCF displays a nearly spherical shape with OCA of 153° in water (Figure 1c), indicating that the bare fabric turned from underwater oleophobicity to underwater superoleophobicity after coating with soil. As shown in Figure S1 (Supplementary Materials), the SCF still preserved superoleophobicity with OCA of 152° under saline water and near superoleophobicity with an OCA of 146° under alkaline water, suggesting the excellent wettability stability of the SCF in saline and alkaline environments. It should be noticed that the SCF cannot be used in acidic environments since the soil coating can react with acid. The oil droplet cannot stick to the surface even it was pressed onto the SCF in water (Figure 1d), and the oil droplets can easily roll off the surface under slightly tilting the fabric (Video S1 in Supplementary Materials), implying the low oil-affinity of the SCF in water. To test the self-cleaning ability of the CF and SCF, they were first soaked in oil-in-water emulsion that was dyed with tony red, and then immersed in water (Figure 1e). It is clear that the CF was contaminated by red oil, while no oil stain was adhered to the surface of SCF, suggesting the excellent self-cleaning capability of the SCF. 

**Figure 2:**
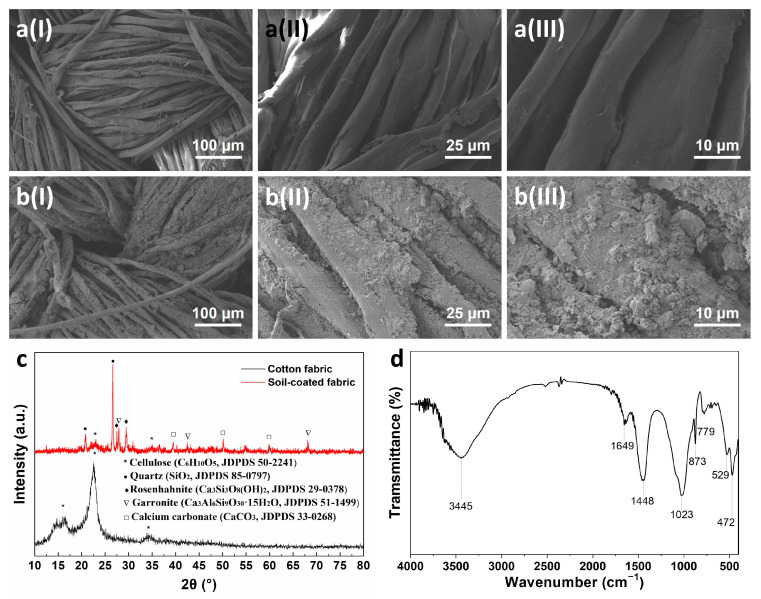
SEM images of the CF (**a**) and SCF (**b**) with different magnifications; XRD patterns of the CF and SCF (**c**); and FTIR spectrum of the SCF (**d**).

Figure 2 displays the morphology of both fabrics. As seen from Figure 2a, the CF is composed of interlaced cotton fibers with a smooth surface. After coating with soil, the fabric still preserves the interwoven structure with micro-scale pores (Figure 2b(I)), which facilitates the rapid permeation of water. High magnification images reveal that the soil nanoparticles are randomly dispersed on the CF surface, forming the micro-scale rough surface (Figure 2b(II,III)). The enhanced roughness on the fabric produced by the nanoparticle coatings could lead to a high value of underwater OCA, which has been proved by previous reports [18]. Such rough microstructure can trap water, which is fundamental for the underwater oil repellency of the SCF.

Figure 2c presents the XRD patterns of the CF and SCF. The CF displays three diffraction peaks of cellulose near 16.02°, 22.65°, and 34.18°, indicating the main component of cellulose of the CF. In addition to the signals of cellulose, the peaks of quartz, rosenhahnite, garronite, and calcium carbonate also appear at the pattern of SCF. The FTIR spectrum of the SCF is displayed in Figure 2d. The absorption peaks at 3445, 1649, and 873 cm^−1^ are attributed to the O-H bonds of rosenhahnite. The peaks at 1023 and 472 cm^−1^ are ascribed to the Si-O vibration of rosenhahnite and garronite. The peaks at 779 and 529 cm^−1^ are associated with the Al-O bonds of garronite. The peak at 1448 cm^−1^ is assigned to the CO_3_^2−^ of calcium carbonate. The abundance of hydroxyl groups and the microstructures endow the SCF with superhydrophilicity.

**Figure 3:**
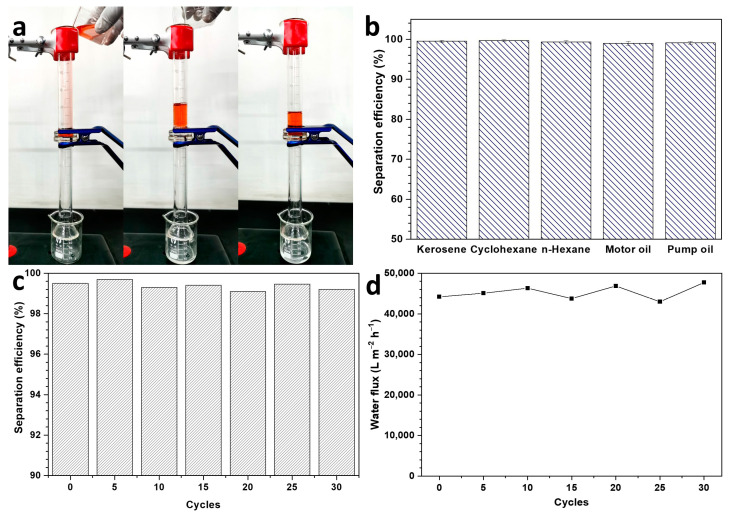
Separation process of kerosene/water mixture (**a**); separation efficiencies of the SCF for different oil/water mixtures (**b**); and variation of the separation efficiency (**c**) and water flux (**d**) for kerosene/water separation as increasing cycle times.

The superhydrophilicity–underwater superoleophobicity suggests that the SCF can be applied as separation membrane for oil/water separation. When the mixture of the kerosene (dyed with tony red) and water was poured into a top tube, the water penetrated through the SCF thoroughly in a few seconds driven by gravity, while the kerosene stayed on the top of the superoleophobic fabric (Figure 3a and Video S2 in Supplementary Materials). The collected water is clear without mixing with visible red kerosene, reflecting that the SCF is available for oil/water separation. In contrast, both oil and water can pass through the CF fixed between the two tubes without selective filtration (Figure S2 in Supplementary Materials). The separation tests were conducted by separating various oil/water mixtures containing kerosene, cyclohexane, n-hexane, motor oil, and pump oil. The separation efficiencies for all mixtures are above 99%, even 99.7% was achieved for the mixture of cyclohexane and water (Figure 3b), implying the outstanding oil/water separation ability of the SCF. These separation efficiencies are higher than most previously reported superhydrophilic/underwater superoleophobic fabrics, such as, cotton fabrics coated with ABC miktoarm terpolymer (97.3% ± 2.5%) [19], cement–sand-coated fabric (>98%) [20], sodium-chlorite-treated CF (>95.7%) [21], polyelectrolyte-coated fabric (>97.3%) [22], and cauliflower-like nickelous hydroxide particles-coated fabric (>96%) [23].

The oil/water separation recyclability of the SCF was assessed by repeating the separation process for 30 cycles. As seen from Figure 3c, the separation efficiencies of the kerosene/water mixture are above 99.1% in the whole separation process, indicating the superior recyclability of the SCF. No soil particle was observed in the collected waters, even under an optical microscope, in the whole recycling process (Figure S3 in Supplementary Materials), reflecting that the soil particles were firmly attached to the fabric. In addition, the SCF still maintains a nearly unchanged ultra-high water flux, of about 45,000 L m^−2^ h^−1^, in all cycles (Figure 3d), suggesting its superior antifouling performance. The ultra-high water flux is much larger than most previously reported superhydrophilic membranes, such as, tannic acid cross-linked laponite-intercalated carrageenan multifunctional membrane (1271 L m^−2^ h^−1^) [24], chondroitin sulfate/poly (acrylamide-co-diallyldimethylammonium chloride)/graphene oxide modified membrane (2316 L m^−2^ h^−1^) [25], cement-sand-coated fabric (2400 L m^−2^ h^−1^) [20], sodium-chlorite-treated CF (2184–4588 L m^−2^ h^−1^) [21], and copper-coated mesh (23,400 L m^−2^ h^−1^) [26]. As shown in Figure S4 (in Supplementary Materials), the morphology of SCF after repeated use for 30 times has no obvious change compared with the original SCF, demonstrating the firm adhesion of soil to the fabric.

## 4. Conclusions

In summary, we present a simple, green, and zero-material cost way to prepare superhydrophilic–underwater superoleophobic SCF through scrape-coating of soil slurry onto CF and subsequent air-drying. The SCF shows an excellent capability for separating various oil/water mixtures with high separation efficiency and recyclability. The SCF also exhibits remarkable antifouling behavior and ultra-high water flux in oil/water separation. The facile production and the superior performance endow the SCFs with promising applications in practical oily water disposal.

## Data Availability

The data presented in this study are available on request from the corresponding author.

## Supplementary Materials

The following supporting information can be downloaded at: https://www.mdpi.com/article/10.3390/membranes13030276/s1, Figure S1: Chloroform droplets on the SCF under 1M NaCl (a) and 1M KOH aqueous solution (b) (inset: underwater OCAs); Figure S2: Separation process oil/water mixture with the original cotton fabric; Figure S3: Optical microscopic photos of the collected waters after 1, 5, 10, 15, 20, 25, 30 times of separation; Figure S4: SEM image of the SCF after oil/water separation for 30 times; Video S1: The rolling off process of oil droplet on the SCF in water; Video S2: The separation process of kerosene/water mixture
