# Supplementary material for: Zero-Material Cost Production of Soil-Coated Fabrics with Underwater Superoleophobicity for Antifouling Oil/Water Separation
Source: Membranes (Basel). 2023 Feb 26;13(3):276. doi: 10.3390/membranes13030276 (PMC10054142; doi:10.3390/membranes13030276)
Supplement: Supplementary file 1 [file membranes-13-00276-s001.zip › Supplementary Information.pdf]

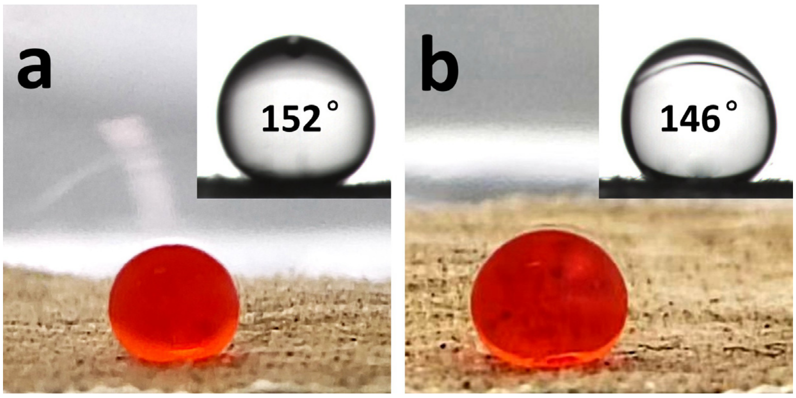

Figure S1. chloroform droplets on the SCF under 1M NaCl (**a**) and 1M KOH aqueous solution (**b**) (inset: underwater OCAs).

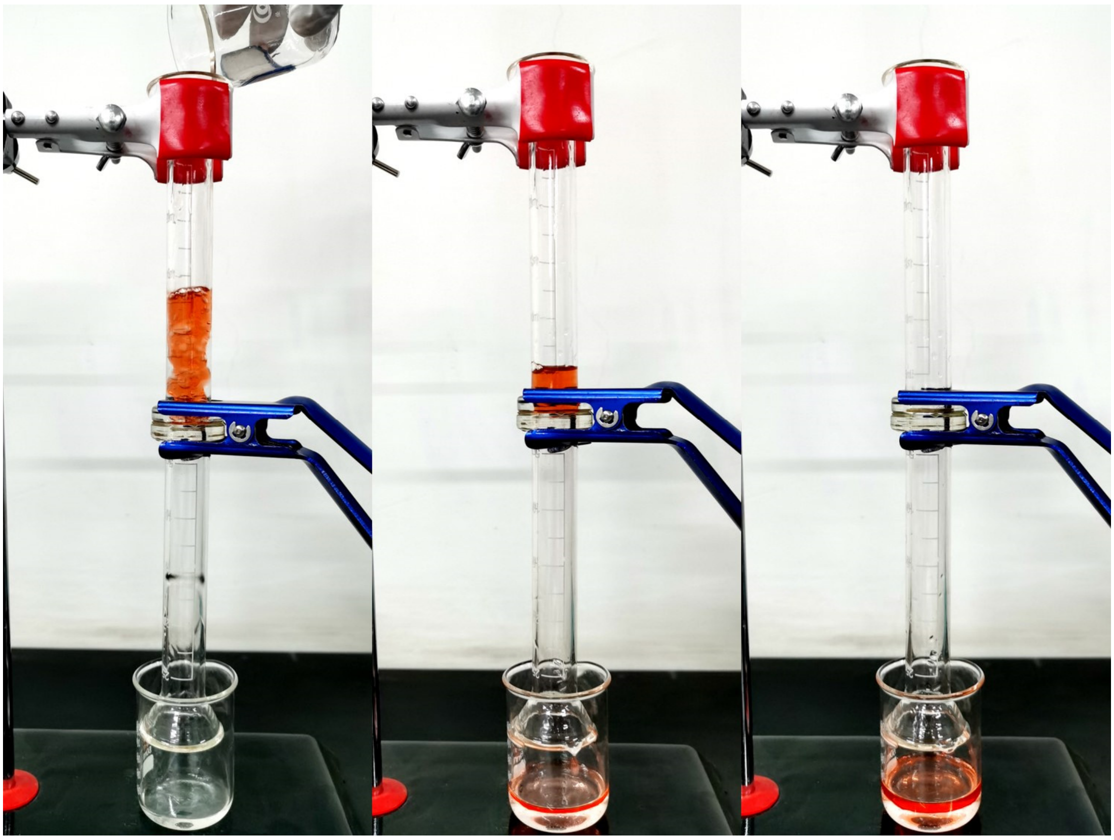

Figure S2. Separation process oil/water mixture with the original cotton fabric.

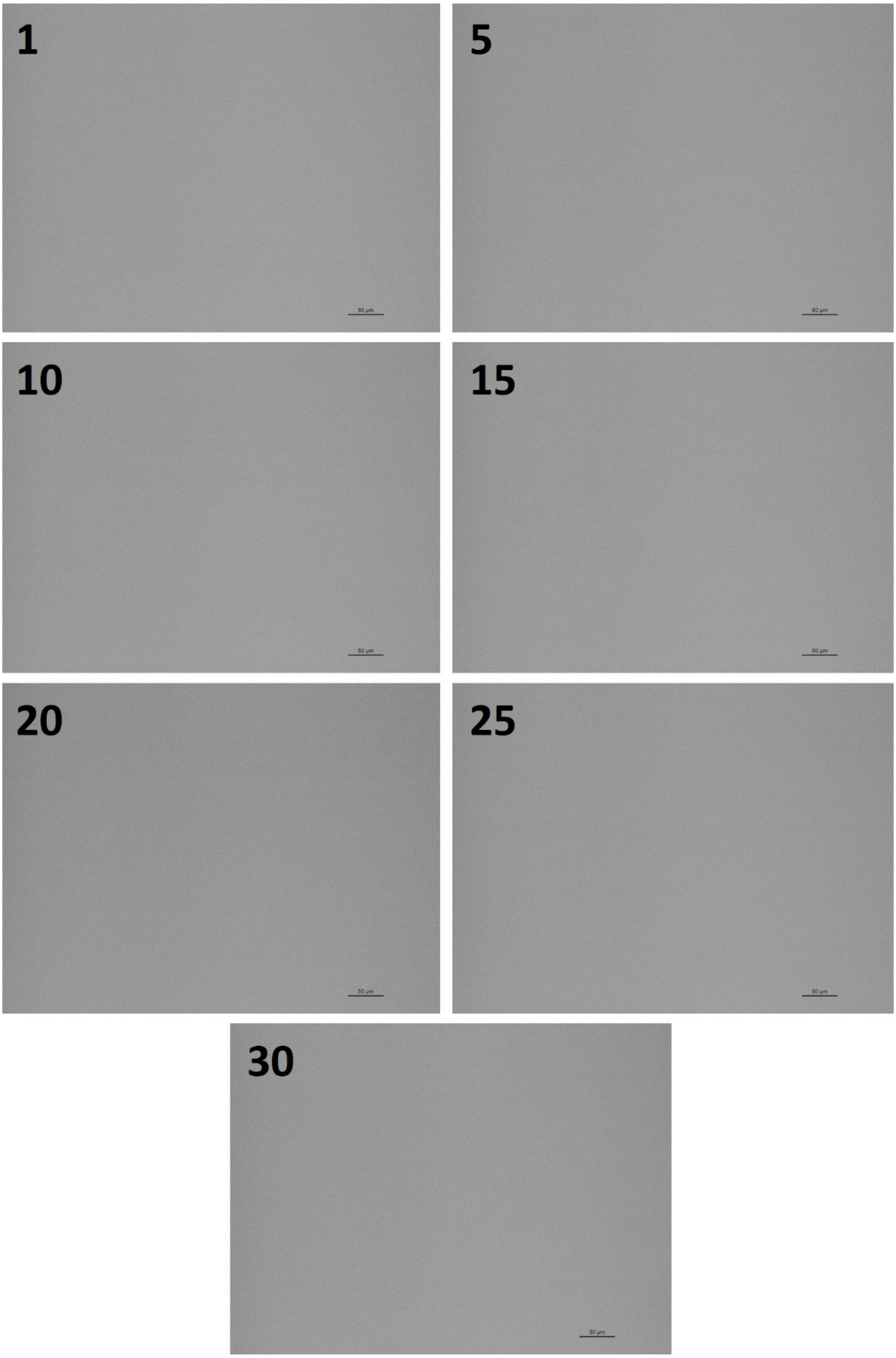

Figure S3. Optical microscopic photos of the collected waters after 1, 5, 10, 15, 20, 25, 30 times of separation.

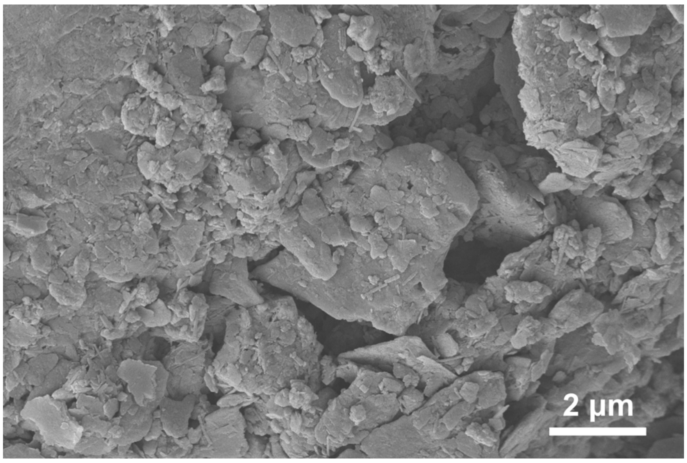

Figure S4. SEM image of the SCF after oil/water separation for 30 times.
